# Editorial

**DOI:** 10.2174/157340312801784925

**Published:** 2012-05

**Authors:** Luke Hermann, Bret P Nelson

In 1924 Willem Einthoven was awarded the Nobel Prize in Medicine for his work in developing electrocardiography, a novel technology that was the first to allow real time analysis of cardiac structure and function. Since that time the variety and sophistication of cardiac testing has skyrocketed, with many modalities currently available to aid clinical decision making 24 hours a day, seven days a week. 

Although this explosion of technology is largely viewed as a net positive for patient care, there are several systems factors which emphasize the need for well-considered and responsible use of these ever more sophisticated resources.

Perhaps most importantly, there is increasing pressure to achieve expeditious and efficient diagnostic results. Whether in the financial form of payment that is attached to “acceptable” hospital or emergency department length of stay or the legal perspective on an “acceptable miss rate” the message is clear. Patients must be evaluated quickly and accurately because to fall out on either metric is to risk financial or legal jeopardy. Thus a delicate balance often exists between cardiac risk stratification that is rapid, cost-effective, and accurate.

At the same time, it has become clear that more testing is not always the answer. Many tests expose patients not only the immediate risk of contrast-related allergic reactions or renal injury but the long term risk of radiation-related cancer. In addition, there is the risk of incidental or false-positive findings that may in themselves lead to medical misadventures. 

In this theme issue of Current Cardiology Reviews, we bring together a series of review articles that specifically address the use of emerging and established diagnostic testing in the evaluation of cardiovascular emergencies. It is our goal to provide the reader a roadmap to the clinical implementation of these ever-expanding technologies. More important, we hope the authors clarify where cardiovascular assessment tools can augment, but not replace, medical decision-making.

